# What Manner of Man Is the Murderer ?

**Published:** 1924-04

**Authors:** 


### WHAT MANNER OF MAN IS THE MURDERER?

### PHYSIQUE AND INTELLIGENCE.

THERE is probably no subject upon which such diversity of opinion exists as the personality of the murderer. Not even within professional circles is there any great measure of agreement, and certainly the world in general has of late had much cause to reflect. In the first place most of us have unhesitatingly concluded that the recent years of war and violence must account indirectly for many a crime. Yet a White Paper published a short time ago totally disproves this contention, a remarkable example of how false a popular impression may turn out to be. Murders have not increased since the war. In 1912 there were in England and Wales 98 supposed murders; in 1913 there were 100. The corresponding figures for 1920 and 1921 are only 107 and 85! In fact, 1921 was an exceptionally good year in this respect. Moreover, if we go into further details it is found that among the number of convictions, the proportion of criminals ultimately declared insane and the number of murderers who committed suicide show an extraordinarily constant level. In short, it is obvious from the official records that neither in frequency of occurrence nor in immunity from detection does the post-war murderer differ very much from his predecessor.

### The Interest of Abnormality.

Nevertheless, although such records dispose of a popular fallacy, they also indicate that we are still lacking in adequate preventive means. Admittedly some murders are committed with a very definite motive by perfectly sane persons, and against these it would seem that our defence lies entirely in force of law and weight of penalties. But the more interesting types of criminals, and they are probably the most frequent, are certainly not of normal mentality. Here it is essentially a matter of early detection of the defective mind. After the crime is committed counsel offers the plea of insanity if that plea is even remotely possible of acceptance. Why is not corresponding activity displayed beforehand? Because of the innumerable difficulties in keeping accurate observations of any free person, let alone a potential murderer, this question needs no detailed answer. Suffice it to say that the study of the criminal mind is being steadily pursued. When some degree of finality is reached it may be possible systematically to watch the most dangerous types.

### The Making of the Criminal.

To realise our present position we have only to consult Dr. Goring's complete and elaborate study of "The English Convict," which has been published by the Government as a Blue Book. His work is the first attempt made in any country to arrive at results in criminology by exact statistical treatment. The main object is to find out to what extent the criminal "make-up," as measured by criminal records, is associated with environment, training, and stock, and with the physical attributes of the criminal. It is possible here to touch but lightly on Dr. Goring's various conclusions, but they are sufficiently important to set going many a train of thought, and when next we discuss the justification of penalties or the "insanity" of a murderer, it may be well to remember these findings.

### No Criminal Type.

As far as head measurements go, it is found impossible to differentiate the criminal from the law-abiding. After examination of innumerable records Dr. Goring finds that "no evidence has emerged confirming the existence of a physical criminal type." Receding foreheads, abnormally shaped ears, colours of hair and eyes give no indication whatever. The criminal classes generally appear to be of somewhat less than average stature, but there the matter ends. Also Dr. Goring is emphatic that there is no such thing as a "mental criminal type." Mental defectives are admittedly liable to commit crime, but, it is argued, unlike the insane and pathological imbeciles, they are not a special class of human beings, but are simply distinguished from other normal persons by their low level of general intelligence. Dr. Goring admits that this inferior intelligence frequently implies an unbalanced mind—a mind that is easily disturbed by impulsiveness, excitability, passionate tempers, &c., but he holds that these qualities are not "morbid," but "natural," being shared in some degree by persons of all mental grades. Therefore, from this analysis, the one significant physical association with crime is defective physique, and the one mental factor is defective intelligence.

### Heredity and Environment.

Let us turn then to heredity. There is evidently a marked tendency for criminal offspring to arise from criminal parents. This may, of course, be due to mere association or to actual heredity, but the figures show that the criminal "make-up" is evidently passed on only with the same frequency as any physical characteristic—the colour of eyes, for example. Then there is the effect of environment. This, too, has, in popular fancy, been considerably exaggerated. Also, does the alcoholic tend to become criminal or the criminal tend to become alcoholic? Or is the relation of alcoholism to crime due to the fact that both have a common antecedent in defective intelligence? It is conclusive from the records that defective intelligence is the fundamental cause—not alcohol as such. It appears incontestable, therefore, that there is no particular type for which to seek. Rather might we say that in a perfect world where the faculties of each, physically and mentally, would be fully and highly developed, the problem of punishment would never arise. The fact that those who fail to live according to our social standards are generally low in the scale of all round development may certainly call into question their whole responsibility for crime, but the fact remains that there is little or nothing, in a medical sense, by which a murderer can be "spotted" before he murders.

